# The 2011 National Wetland Condition Assessment: overview and an invitation

**DOI:** 10.1007/s10661-019-7316-4

**Published:** 2019-06-20

**Authors:** Mary E. Kentula, Steven G. Paulsen

**Affiliations:** Office of Research and Development, National Health and Environmental Effects Laboratory, Western Ecology Division, US Environmental Protection Agency, 200 SW 35th Street, Corvallis, OR 97333 USA

**Keywords:** National Wetland Condition Assessment, National Aquatic Resource Surveys, Wetlands, Monitoring

## Abstract

The first National Wetland Condition Assessment (NWCA) was conducted in 2011 by the US Environmental Protection Agency (USEPA) and its federal and state partners, using a survey design that allowed inference of results to national and regional scales. Vegetation, algae, soil, water chemistry, and hydrologic data were collected at each of 1138 locations across the conterminous United States (US). Ecological condition was assessed in relation to a disturbance gradient anchored by least disturbed (reference) and most disturbed sites identified using chemical, physical, and biological disturbance indices based on site-level data. A vegetation multimetric index (VMMI) was developed as an indicator of condition, and included four metrics: a floristic quality assessment index, relative importance of native plants, number of disturbance-tolerant plant species, and relative cover of native monocots. Potential stressors to wetland condition were identified and incorporated into two indicators of vegetation alteration, four indicators of hydrologic alteration, a soil heavy metal index, and a nonnative plant indicator and were used to quantify national and regional stressor extent, and the associated relative and attributable risk. Approximately 48 ± 6% of the national wetland area was found to be in good condition and 32 ± 6% in poor condition as defined by the VMMI. Across the conterminous US, approximately 20% of wetland area had high or very high stressor levels related to nonnative plants. Vegetation removal, hardening, and ditching stressors had the greatest extent of wetland area with high stressor levels, affecting 23–27% of the wetland area in the NWCA sampled population. The results from the 2016 NWCA will build on those from the 2011 assessment and initiate the ability to report on trends in addition to status. The data and tools produced by the NWCA can be used by others to further our knowledge of wetlands in the conterminous US.

## Introduction

From the time of the Lewis and Clark expedition (1804-06), the United States (US) has a long and rich history of exploring and cataloging our natural resources. Lewis and Clark kept extensive journals on the flora and fauna encountered throughout their expedition, sparking the imagination of others with the rich biological diversity in the American West. Others followed in their footsteps—Wilkes, Pickering, Pike, Whipple, Beckwith, and Fremont, to mention just a few. The fledgling US Geological Survey under John Wesley Powell sponsored numerous surveys and expeditions, as did the Smithsonian Institute’s newly formed National Museum of Natural History. These various surveys and expeditions provided an enormous amount of information on the biological and geological wealth across the US. As the country grew, the focus shifted to the human appropriation of these natural resources for our own use. Eventually, these uses began to border on over use or misuse and required a national response and collaborative effort to protect and restore the quantity and quality of these resources.

In 1972, the US Congress enacted the Federal Water Pollution Control Act, better known as the Clean Water Act (CWA), to protect US water resources. The CWA expresses the national desire to maintain and improve the physical, chemical, and biological integrity of US waters and requires that information on status and trends be reported (Shapiro et al. 2008). The need and desire to improve the quality of water resource assessments is not unique to the US. The international view is exemplified by the Convention on Wetlands, commonly known as the Ramsar Convention, which was signed in 1971 in Ramsar, Iran. The Ramsar Convention was the first modern treaty aimed at conserving natural resources (https://www.ramsar.org/). The more than 162 participating nations work together to halt the loss of wetlands and to promote wise use and management of wetlands through policy making, capacity building, and technology transfer. A specific example of a large-scale effort to protect aquatic systems is the Water Framework Directive 2000/60/EC (WFD) instituted by the European Union (European Commission 2000). The WFD is aimed at facilitating a shift from fragmented policies and approaches to a holistic approach that integrates all parts of aquatic systems (Howarth 2006). However, despite the promise of the establishment of the WFD, after 15 years, the expectations for the WFD have not been realized (Voulvoulis et al. 2017). In another example, the Australia State of the Environment (SOE) reports present the status of the environment with an underlying framework that crosses a number of themes, including inland water and coasts. Beginning in 1996, SOE reports have been published every 5 years. See https://soe.environment.gov.au/ for the 2016 report which contains assessment summaries. The summaries are presented in a searchable report card format that includes items like a change in grade and trend, confidence in results, and comparability to previous years.

A critical section [305(b)] of the CWA calls for periodic accounting to Congress and the American public on the success or failure of efforts to protect and restore US water bodies. Over the past 30 years, multiple groups reviewed the available data and water-quality assessments in the US and concluded that the US Environmental Protection Agency (USEPA) was unable to provide Congress and the public with adequate information regarding the condition of US water bodies (Shapiro et al. 2008). To bridge this information gap, the USEPA, states, tribes, and other federal agencies are collaborating on a monitoring effort to produce assessments that provide the public with improved information. This collaboration resulted in the formation of the National Aquatic Resource Surveys (NARS) (http://www.epa.gov/national-aquatic-resource-surveys) in the early 2000s which assesses the condition of our major water resource types, i.e., estuaries, lakes and reservoirs, and rivers and streams. The last aquatic resource to be included was wetlands, which rounded out the universe of US aquatic resources covered by NARS. This was possible because a series of wetland assessments conducted by USEPA in cooperation with states and other partners had demonstrated that the required technical capability to do wetland assessments at a large scale had been developed (Wardrop et al. 2007; Whigham et al. 2007; Wardrop et al. 2013).

In the recent past, the US federal government has focused primarily on the quantity of wetlands in response to the “no net loss” policy. The policy was established in 1989 by President H.W. Bush as a result of a recommendation of the National Wetland Policy Forum to adopt a goal of no net loss of wetlands in terms of quantity and quality (The Conservation Foundation 1988). The focus on the quantity of wetlands was codified by the 1986 Emergency Wetland Resources Act (Public Law 99–645), which directed the Secretary of the Interior, acting through the US Fish and Wildlife Service (USFWS), to map the wetland resource and to regularly report to Congress on wetland loss (summary by the Congressional Research Service of the Library of Congress at https://www.govtrack.us/congress/bills/99/s740/summary). In response, the USFWS created the National Wetlands Inventory to conduct the mapping and the Status and Trends Program (S&T) to report on the status and trends in wetland area. This approach has provided valuable information since its inception, yet leaves unanswered questions about how we are maintaining and restoring the quality of the wetland resource. Through the National Wetlands Condition Assessment (NWCA), the USEPA is addressing this final aspect of documenting the ecological condition of the aquatic resources of the US.

The NWCA conducted the first national assessment of wetland condition in 2011. It is designed to build upon and augment the achievements of the USFWS S&T program (Dahl 2011). Paired together, the NWCA and S&T provide the public and government agencies tasked with the management of natural resources with comparable, national information on wetland quantity and quality (Scozzafava et al. 2011). The NWCA is designed to produce detailed information on wetland quality by wetland type and region of the conterminous US, thus providing insight on the implications of the changes in area reported by the USFWS S&T program. As stated in the final report of the 2011 assessment (USEPA 2016a), the goals of the NWCA are to:

- “produce a national report describing the ecological condition of the Nation’s wetlands and anthropogenic stressors commonly associated with poor condition;
- collaborate with states and tribes in developing complementary monitoring tools, analytical approaches, and data management technology to aid wetland protection and restoration programs; and
- advance the science of wetland monitoring and assessment to support wetland management needs.” (USEPA 2016a)

This paper contains a summary of the 2011 NWCA methods and results with a discussion of the application of the findings to wetland protection and management. The other papers in the NWCA Topical Collection focus on specific technical aspects of the national assessment. Readers are invited to access and use the NWCA data to pursue additional insights into wetland ecology and management.

## Methods

Below is a brief overview of the methods used in the NWCA abstracted from the 2011 the Site Evaluation Guidelines (USEPA 2011d), Field Operations Manual (USEPA 2011b), Laboratory Methods Manual (USEPA 2011c), and Technical Report (USEPA 2016b).

### Survey design

The 2011 NWCA survey design was linked to the design used by the S&T program by employing the same sample frame to assure production of comparable information on wetland quantity and quality in the US. NWCA and S&T use the following definition of wetlands for which a wetland’s jurisdictional status under state or federal regulatory programs was not a consideration.

“Wetlands are lands transitional between terrestrial and aquatic systems where the water table is usually at or near the surface or the land is covered by shallow water. Wetlands must have *one or more* of the following three attributes:

- at least periodically, the land supports predominantly hydrophytes;
- the substrate is predominantly undrained hydric soil; and/or
- the substrate is non-soil and is saturated with water or covered by shallow water at some time during the growing season of each year (Dahl 2006).”

The 2011 NWCA target population, i.e., the specific portion of the wetlands of the US we aimed to assess, is composed of tidal and nontidal wetlands of the conterminous US, including farmed wetlands not in crop production at the time of the survey, wetlands with rooted vegetation and, when present, open water less than 1 m deep (USEPA 2011b). The target population included seven of the wetland classes used in S&T reporting (Dahl and Bergeson 2009), i.e., estuarine intertidal emergent, estuarine intertidal forested/shrub, palustrine forested, palustrine shrub, palustrine emergent, palustrine unconsolidated bottom/aquatic bed, and palustrine farmed. The classes are an adaptation of those defined by Cowardin et al. (1979).

A spatially balanced probability survey design (Stevens and Olsen 1999, 2000, 2004) was used to generate sufficient sample coordinates (hereafter “points”) to assure a sample size of 900 plus 100 site revisits for quality assurance (USEPA 2016b; Olsen et al. 2019). The NWCA was designed so wetland condition could be reported for the conterminous US by nine aggregated ecoregions (Herlihy et al. 2008) based on the Omernik Level III Ecoregions (Omernik 1987; USEPA 2011a) and by wetland type. The sample was drawn from the USFWS S&T sample frame composed of plots based on 2005 aerial photography and supplemented with additional plotted wetland areas for better coverage on the Pacific Coast. Hereafter, sites from the survey design which were sampled in the field are called “probability sites.”

A total of 1138 sites were sampled in the 2011 NWCA of which 967 were the probability sites used to make the national condition estimates (USEPA 2016b). An additional 21 sites were from a state assessment that did not use the NWCA survey design. The remaining 150 sites were handpicked sites selected to increase the likelihood of identifying high-quality reference sites. These “nonprobability sites” cannot be used to make national condition estimates but were used with the probability sites to establish a disturbance gradient and identify reference sites (USEPA 2016b; Herlihy et al. 2019a).

**Fig. 1:**
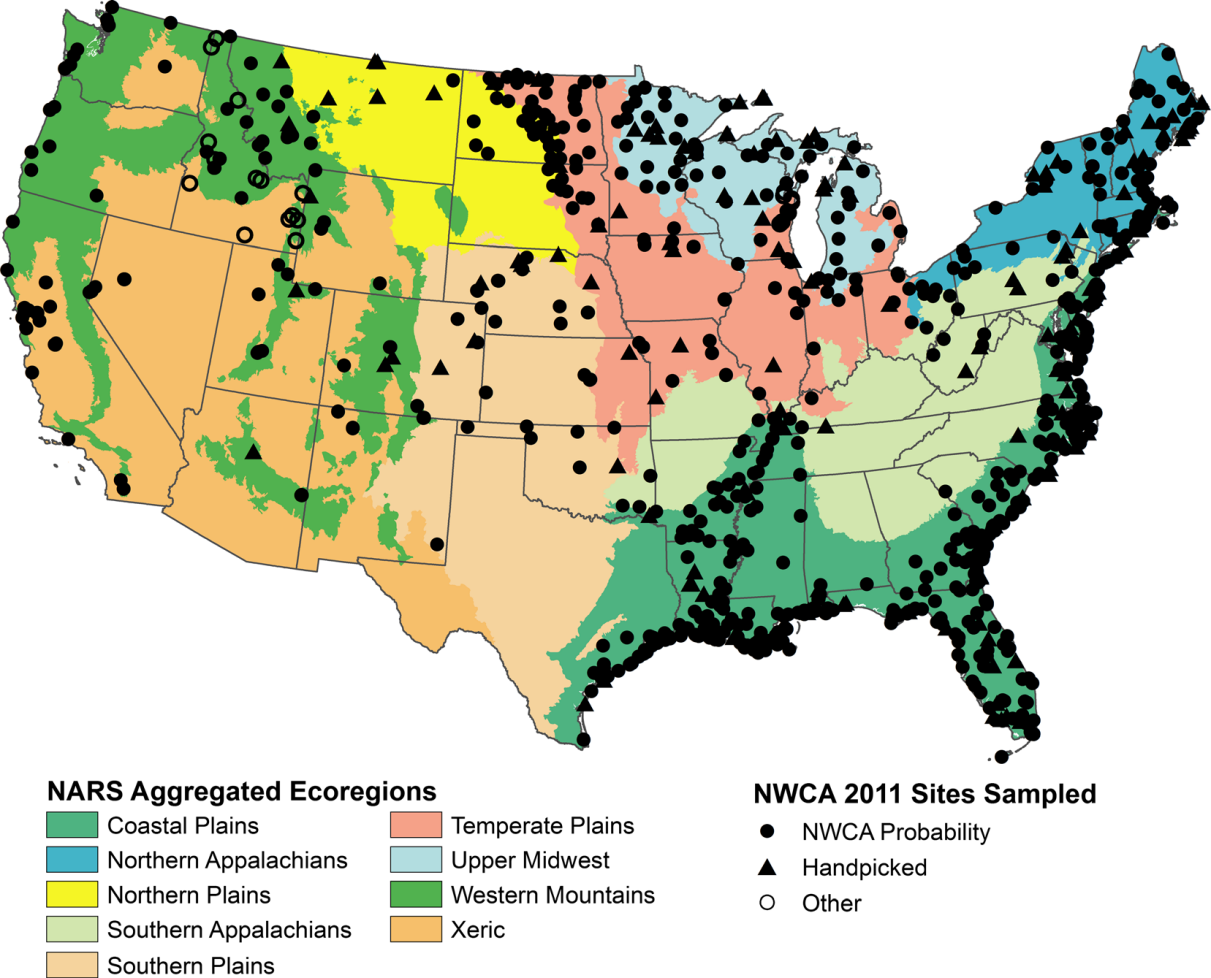
Map of the conterminous United States showing the distribution of the 1138 sites sampled from the National Wetland Condition Assessment (NWCA), which included sites from the probability design, the handpicked sites, and sites from other sources (*adapted from* USEPA 2016b). The nine National Aquatic Resource Survey (NARS) Aggregated Ecoregions are a combination of the Level III ecoregions (Omernik 1987) used in site selection for the 2011 NWCA and in other NARS assessments (Herlihy et al. 2008)

The sites were distributed throughout the conterminous US (Fig. 1). The spatial distribution in the probability sites follows the distribution of wetlands as represented in the S&T sample frame, as influenced by the pattern of access denial in a region. The sites sampled represent the inference population of 62.2 ± 5.28 million acres of wetland area and approximately 65% of the target population (94.9 ± 6.20 million acres) (USEPA 2016b; Olsen et al. 2019).

### Field sampling

**Fig. 2:**
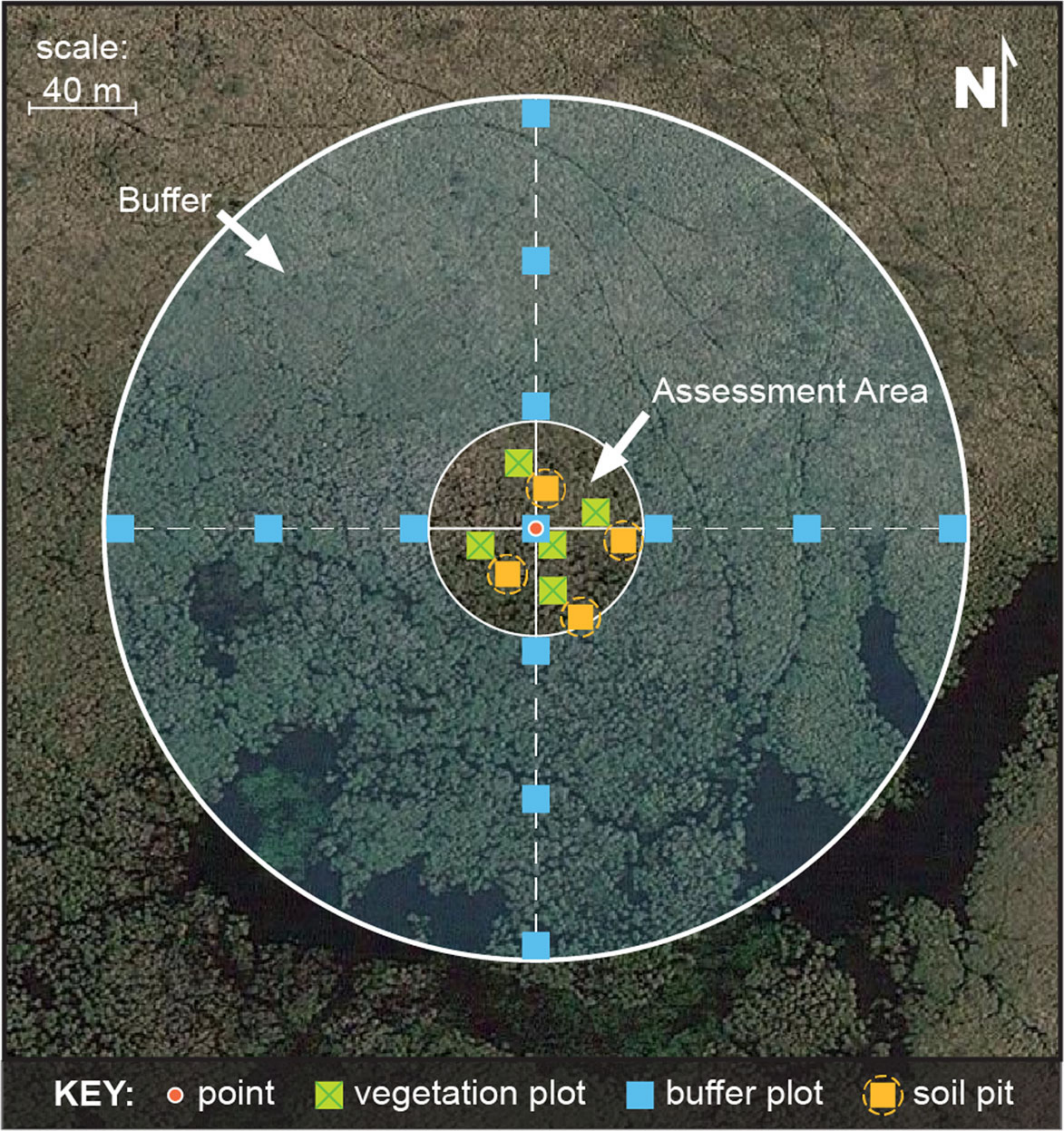
Diagram of a standard layout for a 0.5-ha assessment area and a surrounding 100-m buffer (*adapted from* USEPA 2016b). Locations of the point from the survey design and of the sampling done in plots are indicated

NWCA protocols were designed to be completed by a four-person field crew during one field-day (USEPA 2011b; McCauley et al. 2019). The crew sampled an assessment area (AA) and an area immediately adjacent to the AA (i.e., the buffer). The standard AA was a ½-ha circular plot with a 40-m radius, centered on the location of the point with the buffer extending 100 m from the edge of the AA (Fig. 2). Alternate configurations for AA size and shape and the location of sample locations were adjusted in relation to specific site conditions using a rule-based system (USEPA 2011b). The indicators used in the analysis and a brief description of the sampling approach for each is presented below; the detailed protocols are found in the NWCA Field Operations Manual (USEPA 2011b).

Vegetation data were used to create the NWCA’s indicator of biological condition and a nonnative plant indicator (USEPA 2016b; Magee et al. 2019a, b). Data were collected during the peak growing season when most plants are in flower or fruit to optimize species identification and for characterization of species abundance. In addition, data on vegetation structure were collected. Data were gathered in five, systematically placed, 100-m^2^ vegetation plots within the AA (Fig. 2) (USEPA 2011b).

Physical and chemical data were collected to generate indicators of stressors potentially impacting wetland condition. Evidence of human activities in the AA and buffer was based on observational data collected from 13 10 m × 10 m plots (one in the center of the AA; 12 in the buffer) (Fig. 2) (USEPA 2011b; Lomnicky et al. 2019 ). Soil chemistry was measured in one of four pits (Fig. 2) (USEPA 2011b) designated as representative of the AA by the field crew. Soil samples were collected from each layer to a depth of 125 cm and analyzed for heavy metals and phosphorus by the US Department of Agriculture, Natural Resource Conservation Service, Kellogg Soil Survey Laboratory, Lincoln, NE, according to standard procedures (USEPA 2011c; Nahlik et al. 2019).

### Analysis

The master database for the 2011 NWCA contains several types of data. First, there are the raw data collected in the field and laboratory. Data characterizing the NWCA sites include site information from the survey design and ancillary information on biological traits and from GIS layers. Raw data, information from the design, and ancillary data are used separately or combined into metrics for specific analyses.

**Fig. 3:**
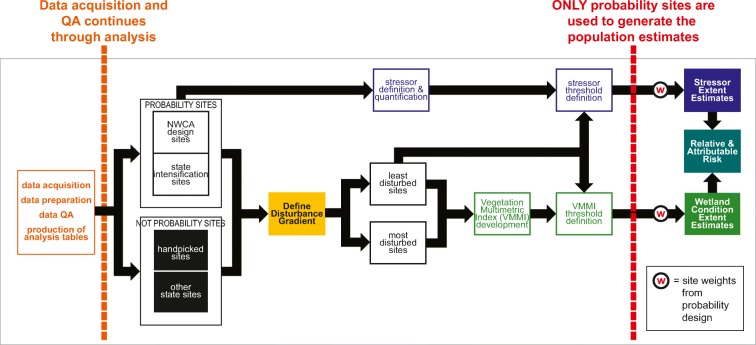
Flowchart showing the major components of the analysis for the National Wetland Condition (*adapted from* USEPA (2016b))

The analysis involved a number of interrelated tasks composed of multiple steps. Figure 3 illustrates four key components: (1) quality assurance; (2) disturbance gradient establishment; (3) index development; and (4) population estimates, which are described in the following sections (USEPA 2016b).

### Quality assurance

Three types of quality assurance (QA) checks were completed before datasets were assembled for analysis: (1) verification of the sampling status of every point considered for sampling; (2) confirmation of longitudes and latitudes associated with the sites sampled; and (3) data checks (Fig. 3) (USEPA 2016b).

All points from the design were reviewed to confirm sampling or to confirm a documented reason for not being sampled. The review was performed using information: (1) compiled during pre-sampling evaluation of points; (2) recorded during a field evaluation performed prior to sampling; and (3) recorded at the time of sampling (USEPA 2011d).

Longitudes and latitudes were measured at various key locations associated with field sampling, and in particular, at the location of the point from the design. These coordinates were especially important if a point needed to be relocated or shifted to accommodate the sampling protocol (USEPA 2011b). The coordinates were used to (1) verify the relationship between the location of the point from the design and the AA; (2) tie the field data to landscape data from GIS layers; and (3) relocate the site and key locations of the field sampling protocol (e.g., the AA center, vegetation plots) for resampling in future surveys (USEPA 2016b).

Point coordinates from the design and the field were compared. The locations of points from the field that were more than 60 m from the corresponding design coordinates, i.e., that exceeded protocol guidelines, were flagged. There were 25 sites that required further evaluation, but all 25 sites were determined to meet design standards because (1) permission to move the point beyond 60 m was granted because the proposed AA center met design specifications, (2) recording errors made by the field crew were identified and corrected, or (3) the distance exceeding 60 m from the sample point was determined to be negligible (USEPA 2016b).

The R Statistical package (R Core Team 2015) was used to query the raw data and generate a list of missing data and to identify why these data were missing (e.g., not collected by the field crew, data not entered on the field or lab form). Additional R code was written for each data type to generate a list of data not meeting specific legal value and range tests. These tests confirmed that the data type was correct, data fell within the valid range or legal values, and units reported matched those expected (USEPA 2016b).

Results of the checks that did not agree with the QA data requirements were evaluated, often by referring to the original forms submitted by the field crew or laboratory (USEPA 2016b). A description of the error and a recommended resolution were recorded for each data type and incorporated into the master NWCA database as metadata when data corrections were implemented. The analysis lead for each data type was consulted in cases where the resolution of the issue was ambiguous and could affect the interpretation of results.

### Arraying the sites sampled along a disturbance gradient

NARS assessments employ a disturbance gradient for developing and testing metrics and indices used to report on biological condition and on the stressors potentially impacting the biota. A disturbance gradient is created for each reporting unit that reflects the level of disturbance documented. The steps in establishing the gradient are to (1) define reporting units; (2) identify disturbance data to be used to screen sites for placement along a disturbance gradient; and (3) set thresholds for least (i.e., reference) and most disturbed for each disturbance index or metric to establish the ends of the gradient (USEPA 2016b; Herlihy et al. 2019a).

The NWCA preference for reporting is to describe the results by wetland types and ecoregions. USEPA’s Environmental Monitoring and Assessment Program (EMAP) recommended as a general rule that, without specific information on the variability within the target population, 50 sites *per reporting unit* should be assessed to increase the likelihood that the sample size will be sufficient to make population estimates with reasonable confidence intervals (see www.epa.gov/nheerl/arm/surdesignfaqs.htma and (Herlihy et al. 2008)). The combination of the nine aggregated ecoregions and seven wetland types used in the NWCA survey design resulted in 56 potential reporting groups, most of which had fewer than 50 sampled sites. The 56 potential reporting groups were aggregated based on a series of ordinations evaluating the relationship between plant species composition, ecoregion, and wetland type (USEPA 2016b; Herlihy et al., 2019a). Vegetation data were used because vegetation is the NWCA indicator of biological condition. The goal was to maximize within-group similarity in plant communities while creating groups useful in reporting assessment results (USEPA 2016b). The analyses resulted in four NWCA Aggregated Ecoregions and four NWCA Aggregated Wetland Types which were combined to produce 10 reporting groups (Table 1).

**Table 1:** Matrix of the four NWCA Aggregated Ecoregions (left column) and the four NWCA Aggregated Wetland Types (top row) used to form the 10 NWCA Reporting Groups (body of the matrix) (*adapted from* USEPA (2016b)

NWCA Aggregated Ecoregions	NWCA Aggregated Wetland Types			
	Palustrine, riverine, and lacustrine herbaceous (PRLH)	Palustrine, riverine, and lacustrine woody (PRLW)	Estuarine herbaceous (EH)	Estuarine woody (EW)
Coastal Plains (CPL)	Coastal Plains herbaceous (CPL-PRLH) *n* = 72	Coastal Plains woody (CPL-PRLW) *n* = 189		
Eastern Mountains and Upper Midwest (EMU)	Eastern Mountains and Upper Midwest herbaceous (EMU-PRLH) *n* = 73	Eastern Mountains and Upper Midwest woody (EMU-PRLW) *n* = 127		
Interior Plains (IPL)	Interior Plains herbaceous (IPL-PRLH) *n* = 138	Interior Plains woody (IPL-PRLW) *n* = 52		
West (W)	West herbaceous (W-PRLH) *n* = 67	West woody (W-PRLW) *n* = 75		
National (ALL)			Estuarine herbaceous (ALL-EH) *n* = 272	Estuarine woody(ALL-EW) *n* = 73

Note estuarine reporting groups are formed nationally (ALL) and not by ecoregion due to sample size constraints

Disturbance data collected from the 1138 sites sampled were evaluated for utility in screening sites for placement along a disturbance gradient. Types of disturbance data were chosen based on evidence of a strong association with anthropogenic stress and on the robustness of the data (USEPA 2016b). Nine indices and one metric were developed within four categories of disturbance and were used to screen each site. The ten screens represented:

- Disturbance in the Buffer and AA (six indices developed) (USEPA 2016b; Lomnicky et al. 2019),
- Hydrologic alteration in the AA (two indices developed) (USEPA 2016b; Lomnicky et al. 2019),
- Soil chemistry in the AA (one index developed) (USEPA 2016b; Nahlik et al. 2019), and
- Relative cover of alien plant species in the AA (one metric developed) (USEPA 2016b; Magee et al. 2019b).

Thresholds for each of the ten screens were set independently for each of the NWCA Reporting Groups because type and frequency of human disturbance can vary greatly among ecoregions and wetland types. As described in the NWCA Technical Report (USEPA 2016b):

“A disturbance gradient was defined by categorizing NWCA sites into least, intermediate, or most disturbed categories. Initially, thresholds for the least disturbed category were set to zero to reflect minimal (i.e., no observable) human disturbance with the exception of a threshold of >5% relative cover for the alien plant species metric. If a Reporting Group had a sufficient number of sites passing all thresholds (i.e., approximately 15-25% of the sites in a Reporting Group), then a threshold of zero was used to identify least disturbed sites. If an insufficient number of sites met the criteria for least disturbed (i.e., minimal human disturbance), the thresholds were relaxed from zero to obtain a sufficient number of sites in the least disturbed category.”

**Fig. 4:**
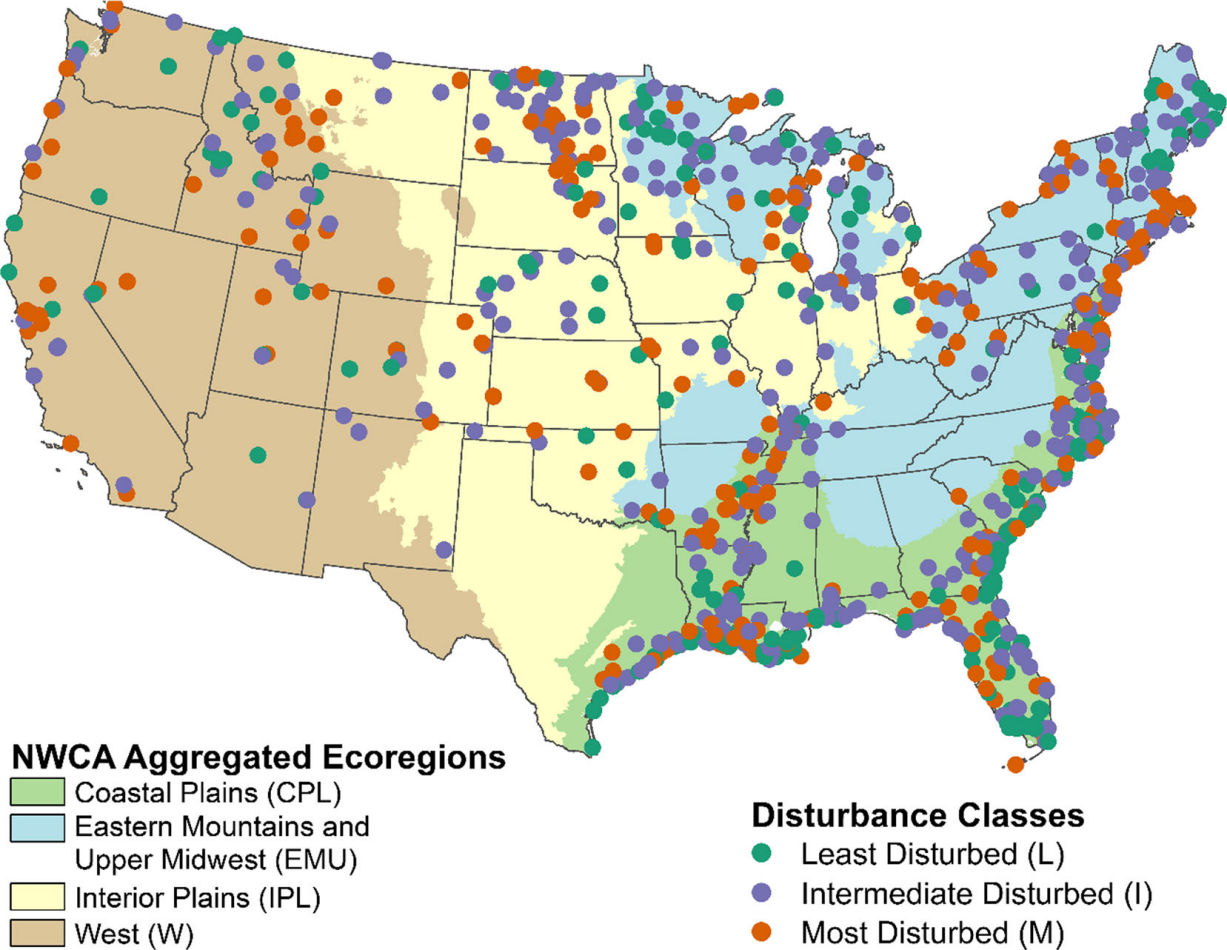
Distribution of 2011 NWCA sites sampled by disturbance category (*from* USEPA (2016b)

Most disturbed sites were defined using a screening process similar to the one used to define least disturbed sites. The same ten screens were used, and thresholds for most disturbed were set by Reporting Group. If the threshold for any measure of disturbance was exceeded, the site was considered a most disturbed site. The objective was to define approximately 20–30% of the sites in a Reporting Group as most disturbed and thresholds were set accordingly (USEPA 2016b). Sites not falling into either least or most disturbed were classified as intermediate disturbed (Fig. 4).

### Development of indicators of condition and stress

The NWCA reports on ecological condition and the extent of stressors at national and regional scales using biological, chemical, physical, and hydrologic indicators, which are described below.

Vegetation data were used to construct a Vegetation Multimetric Index (VMMI) as the indictor of wetland condition (Fig. 3) (USEPA 2016b; Magee et al. 2019a). Natural vegetation has been increasingly used as an indicator of ecological condition in wetlands (Mack and Kentula 2010) because of the relationship between disturbance and shifts in plant species, functional groups, e.g., Quétier et al. (2007), communities, e.g., DeKeyser et al. (2003), and vegetation structural elements, e.g., Mack (2007). An approach developed by van Sickle (2010) was adapted to calculate and evaluate thousands of potential VMMIs to identify those with the highest performance. The final national-scale VMMI was composed of four components (Table 2). Thresholds delineating good, fair, and poor condition were based on the distribution of VMMI values in least disturbed sites (Stoddard et al. 2006) in each of the 10 ecoregion by wetland type Reporting Groups (Table 1).

**Table 2:** Description and components of the Vegetation Multimetric Index and the indicators of stress (*adapted from* USEPA (2016b)

Indicators	Description	Items included
Indicator of biological condition		
Vegetation Multimetric Index (VMMI)	A four metric, national-scale VMMI was selected as having the best overall performance in assessing wetland condition based on a series of objective screening criteria	Floristic Quality Assessment Index, relative importance of native plants (combines relative cover and relative frequency of native plants), number of species tolerant to disturbance, relative cover of native monocots
Indicators of stress		
Biological indicator		
Nonnative plant indicator (NNPI)	Composed of three metrics that describe different avenues of potential impact to biological condition	Relative cover of nonnative species, richness of nonnative species, relative frequency of nonnative species
Physical indicators		
Vegetation removal	Any field observation related to loss, removal, or damage of wetland vegetation	Gravel pit, oil drilling, gas wells, underground mine, forest clear cut, forest selective cut, tree canopy herbivory, shrub layer browsed, highly grazed grasses, recently burned forest, recently burned grassland, herbicide use, mowing/shrub cutting, pasture/hay, range
Vegetation replacement	Any field observation of altered vegetation within the site due to anthropogenic activities	Golf course, lawn/park, row crops in small amounts in the assessment area, row crops in the buffer, fallow field, nursery, orchard, tree plantation
Damming	Any field observation related to impounding or impeding water flow from or within the site	Dike/dam/road/RR bed, water level control structure, wall/riprap, dikes, berms, dams, railroad beds, sewer outfalls
Ditching	Any field observation related to draining water	Ditches, channelization, inlets/outlets, point source/pipe, irrigation, water supply, field tiling, standpipe outflow, corrugated pipe, box culvert, outflowing ditches
Hardening	Any field observation related to soil compaction, including activities and infrastructure that primarily result in soil hardening	Gravel road, two-lane road, four-lane road, parking lot/pavement, trails, soil compaction, off road vehicle damage, confined animal feeding, dairy, suburban residential, urban/multifamily, rural residential, impervious surface input, animal trampling, vehicle ruts, roads, concrete, asphalt
Filling/erosion	Any field observation related to soil erosion or deposition	Excavation/dredging, fill/spoil banks, freshly deposited sediment, soil loss/root exposure, soil erosion, irrigation, landfill, dumping, surface mine, recent sedimentation, excavation/dredging
Chemical indicators		
Heavy Metal Index	Heavy metals with concentrations above background concentrations in soil samples	Antimony, cadmium, chromium, cobalt, copper, lead, nickel, silver, tin, tungsten, vanadium, zinc concentrations from the uppermost layer with soil chemistry
Soil phosphorus concentration	Soil phosphorus concentrations relative to reference sites	Measured phosphorus concentration from the uppermost layer within 10 cm of the soil surface with soil chemistry

Different approaches were used to develop indicators of biological, physical, and chemical stress (Fig. 3). The nonnative plant indicator (NNPI) was developed as the biological indicator of stress (USEPA 2016b; Magee et al. 2019b). The NNPI is composed of three metrics describing potential effects of the complement of nonnative taxa occurring at each site (Table 2). The NNPI was used to define four stressor-level categories (low, moderate, high, and very high) based on exceedance thresholds for any one of the three component metrics (USEPA 2016b; Magee et al. 2019b).

Field crews recorded observations of 52 pre-defined human activities encountered in the AA and buffer (USEPA 2011b). Hydrologic alterations observed within an AA were tallied as were observations of human activities in 13 plots (12 in the buffer and one at the center of the AA). These data were regrouped into six stressor categories: vegetation removal, vegetation replacement, damming, ditching, hardening, and filling/erosion (Table 2). An Anthropogenic Stress Index (ASI) was developed for each of the six stressor categories and thresholds for low, medium, and high stressor-levels were established (USEPA 2016b; Lomnicky et al. 2019). Each site was assigned to a stressor-level for each of the six stressor categories based on its ASI score.

Soil chemistry data were examined to identify chemical indicators of stress and only heavy metals and phosphorus were ultimately used (Table 2). Twelve heavy metals, each (1) with high signal to noise ratios (Kaufmann et al. 2014), (2) a close relation to anthropogenic impacts, and (3) occurring in consistently measurable quantities, were used to develop a Heavy Metals Index (HMI) (USEPA 2016b; Nahlik et al. 2019). The metals were silver, cadmium, cobalt, chromium, copper, nickel, lead, antimony, tin, vanadium, tungsten, and zinc. The HMI is the sum of the number of metals present in the uppermost layer with soil chemistry at a site with concentrations above natural background levels based on published values directly or slightly modified from primarily Alloway (2013; USEPA 2016b). In the case of phosphorus, the value for the soil phosphorus concentration for the uppermost layer with soil chemistry was used as a chemical indicator of stress. Because no published thresholds for anthropogenic impacts to wetlands were available, thresholds for chemical stressor-levels were set using best professional judgment (USEPA 2016b). The low stressor-level threshold was set to zero, i.e., all metals were less than or equal to background concentrations. The threshold for the high stressor-level was ≥ 3 metals above background. All values falling between the high and low-stressor levels were termed moderate. For phosphorus, the thresholds for low and high-stressor levels were set using the 75th and 95th percentiles observed in least disturbed sites (Herlihy et al. 2008; Herlihy et al. 2013).

### Population estimates

Estimates of the wetland area falling into a particular condition class are based on the weights from the survey design (Fig. 3). For detailed examples of how this has been done, see Stevens and Jensen (2007), Olsen and Peck (2008), and Olsen et al. (2019). The weight indicates the wetland area in the NWCA target population represented by a point from the sample draw. After the assessment was conducted, the weights were adjusted to account for additional points used when primary points could not be sampled (e.g., due to denial of access, site not a wetland). The weights and inference algorithms were then used to calculate wetland biological condition, stressor extent, and relative and attributable risk (Van Sickle and Paulsen 2008) expressed as estimates of wetland area (i.e., numbers of acres or percent of the entire resource) in a particular condition class or stressor-level for the NWCA sampled population across the conterminous US and by NWCA Aggregated Ecoregion and NWCA Aggregated Wetland Type (USEPA 2016b).

## Results and discussion

Results are reported for the Nation, i.e., the conterminous US, and by NWCA Aggregated Ecoregion and NWCA Aggregated Wetland Type. For each, biological condition and stressor extent, relative extent, and relative and attributable risk are presented. See the final and technical reports for the 2011 NWCA (USEPA 2016a, b) for additional details which are abstracted here.

### Condition of wetlands for the Nation, ecoregions, and wetland types

**Fig. 5:**
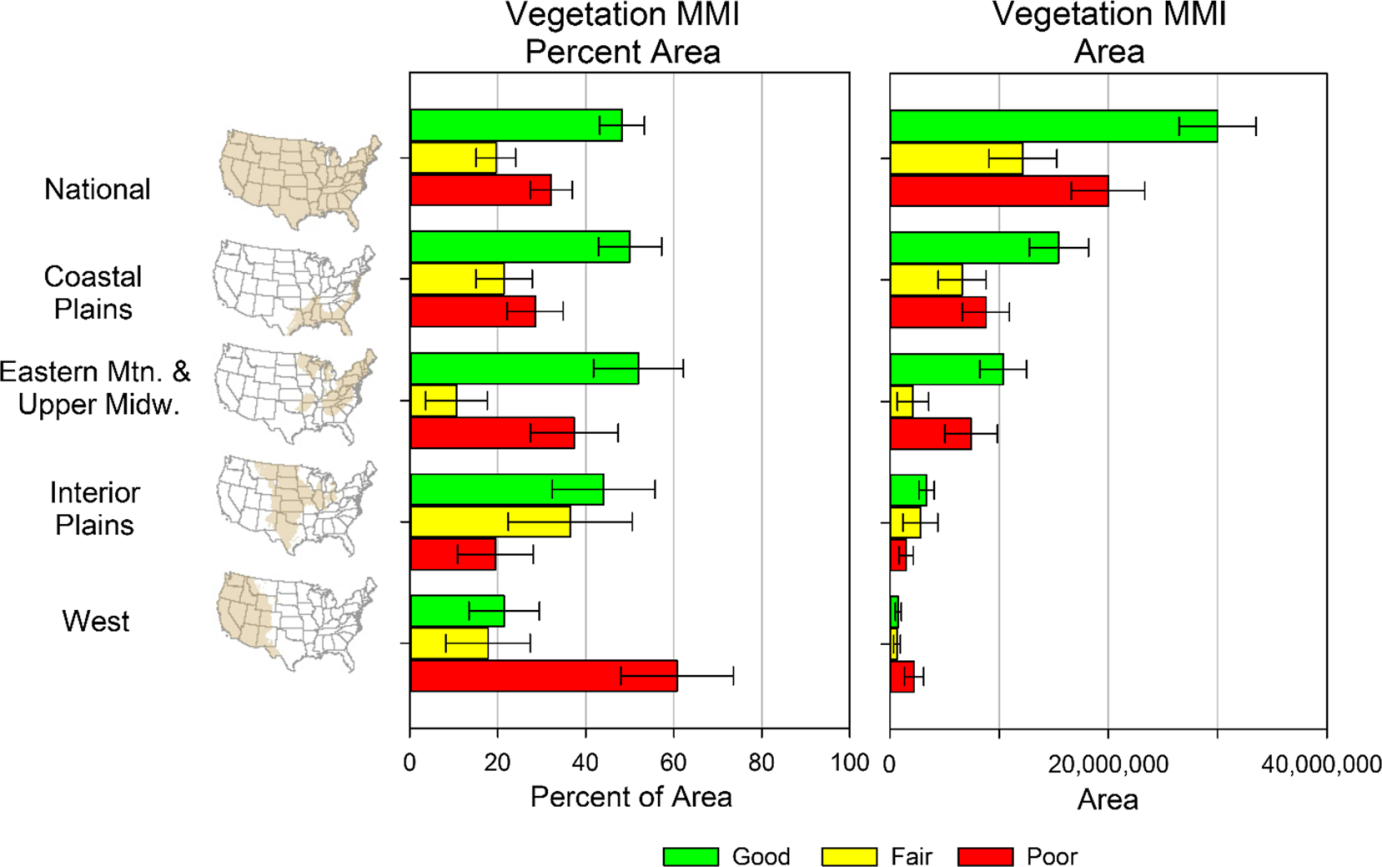
Estimated wetland biological condition by class (good, fair, poor) and area (acres) for the Nation and the NWCA Aggregated Ecoregions (Table 1). Error bars are 95% confidence intervals (*from* USEPA (2016a)

The biological condition of the wetlands in the conterminous US was determined using the VMMI. The 2011 NWCA found 48% of the national wetland area was in good condition, 20% in fair condition, and 32% in poor condition (Fig. 5) (USEPA 2016a). The national pattern of biological condition reflects the results for the NWCA Aggregated Ecoregions with the greatest wetland area, i.e., Coastal Plains (CPL), and the Eastern Mountains and Upper Midwest (EMU) (Fig. 5). The pattern varies for the Interior Plains (IPL) and West (W), with the W being the most different with 61% of its wetland area in poor condition versus 32% nationally. It is important to note that although the results in the IPL and W represent a small proportion of the wetland area nationally, the results represent a large portion of the wetland area in each of these regions (USEPA 2016a).

**Fig. 6:**
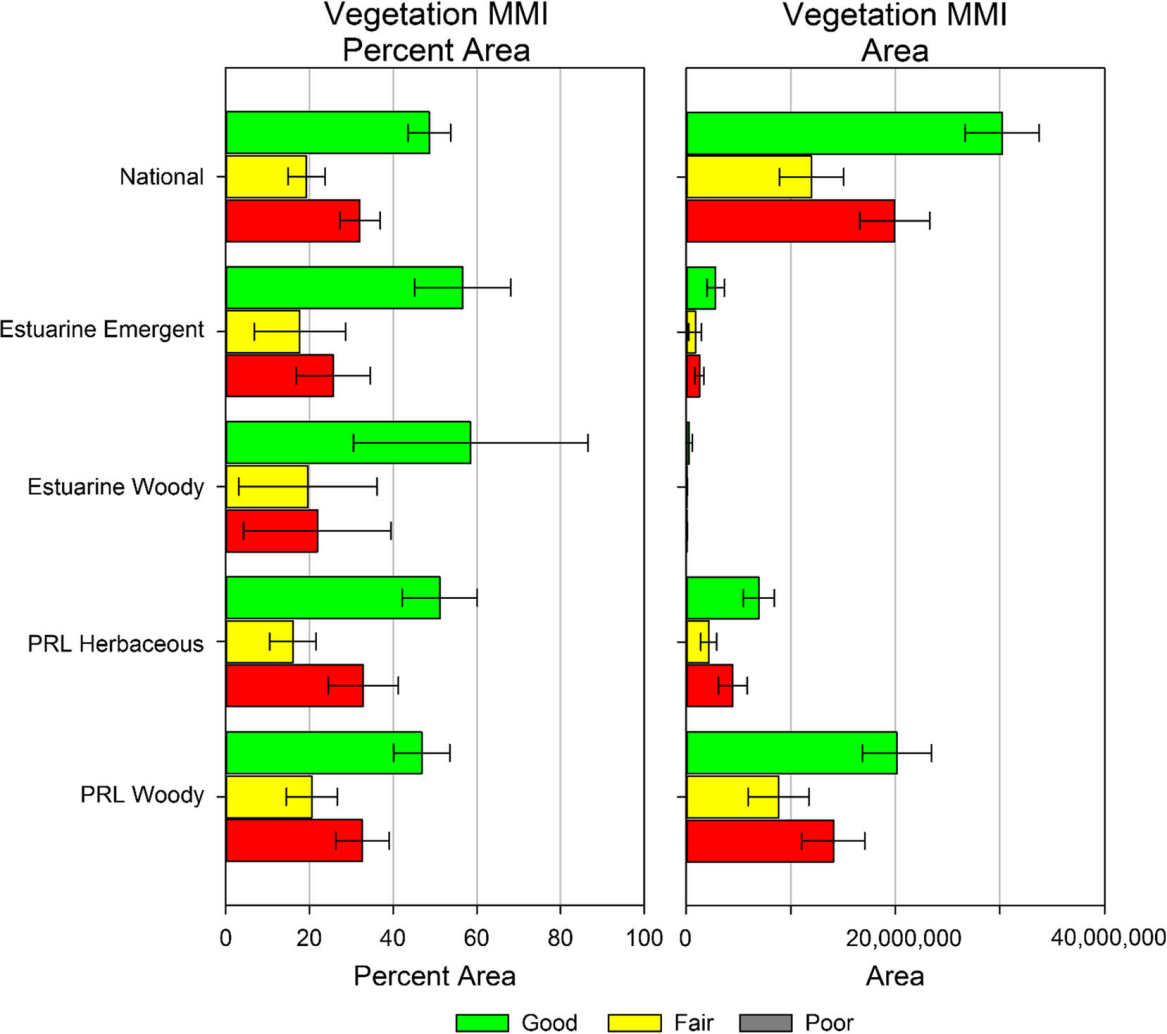
Estimated wetland biological condition by class (good, fair, poor) and area (acres) for the Nation and NWCA Aggregated Wetland Types (Table 1). Error bars are 95% error intervals. PRL = palustrine, riverine, lacustrine (*from* USEPA (2016a)

The biological condition of the wetlands in the conterminous US by NWCA Aggregated Wetland Type mirrors the national results (Fig. 6) with around 50% of the area in good condition (USEPA 2016a). There were no clear differences in condition between wetland types. However, the national-scale results were likely influenced by the results from the woody and herbaceous palustrine, riverine, and lacustrine (PRL) wetland types, which have the largest sample sizes and wetland area across the conterminous US (USEPA 2016a).

### Assessment of stressors

Understanding the relative magnitude or importance of potential stressors to wetlands across the area of interest is essential to making policy and management decisions. Both the prevalence (i.e., extent of wetland area with high levels of a stressor) and the severity of each stressor (i.e., influence on biological condition in relation to the influence of other stressors) must be considered (Van Sickle and Paulsen 2008). To provide such information on stressors, separate rankings of the relative extent and the associated relative and attributable risk to US wetlands were generated for the NWCA (USEPA 2016b).

### Stressor extent

Stressor extent is an estimate of how spatially common an indicator of stress is nationally or regionally. The 2011 NWCA evaluated nine different indicators of stress, including six physical indicators (USEPA 2016a, b; Lomnicky et al. 2019), two chemical indicators (USEPA 2016a, b; Nahlik et al. 2019), and one biological indicator (USEPA 2016a, 2016b; Magee et al. 2019b).

**Fig. 7:**
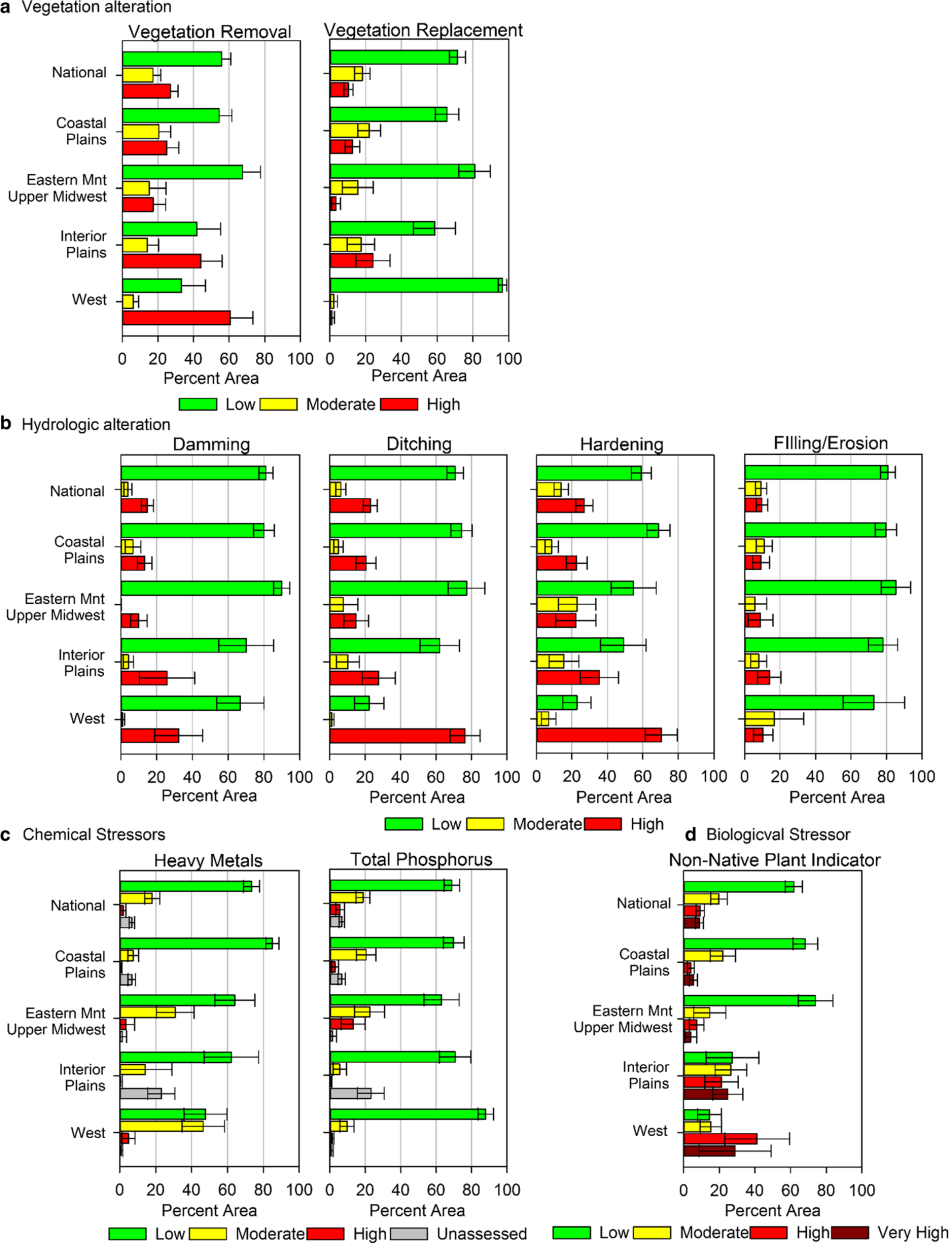
Estimated extent of wetland area (acres) affected by stressor-level for physical (**a**, **b**), chemical (**c**), and biological (**d**) indicators of stress for the Nation and NWCA Aggregated Ecoregions (Table 1). Error bars are 95% confidence intervals (*adapted from* USEPA (2016a)

The pattern of extent for most physical indicators of stress showed the highest percent of wetland area at low stressor-levels followed by high-stressor levels (Fig. 7) (USEPA 2016a; Lomnicky et al. 2019). The exception was the W, where the greatest percent wetland area with high stressor levels was for vegetation removal, ditching, and hardening. Hardening includes paved surfaces like roads but also severe soil compaction due to activities like vehicle use, foot traffic, and/or livestock grazing. This pattern also supports the finding that a high percentage of wetland area in the W ecoregion is in poor biological condition (USEPA 2016a).

Chemical stressors in the soil were assessed using a Heavy Metal Index and soil phosphorus levels (Fig. 7) (USEPA 2016b; Nahlik et al. 2019). At least 60% of the wetland area in the estimated wetland area at the national-scale and CPL, EMU, and IPL ecoregions had low stressor-levels for heavy metals (USEPA 2016a). Again, the W was the exception with low and moderate stressor-levels each affecting about 50% of the wetland area in the region. In the case of total soil phosphorus, the Nation and all NWCA Aggregated Ecoregions had ≥ 63% of their area with low stressor-levels (USEPA 2016a).

Stressor-levels for nonnative plants were low across a large percent of estimated wetland area at the national scale (61%) and for the CPL (66%) and EMU (74%) aggregated ecoregions (Fig. 7) (USEPA 2016a; Magee et al. 2019b). In the IPL and W ecoregions, the extent of wetland area with low stressor-levels for nonnative plants was smaller at 27% and 14%, respectively. The W had a noteworthy percent of area at high (42%) and very high stressor-levels (30%).

### Estimating risk

**Fig. 8:**
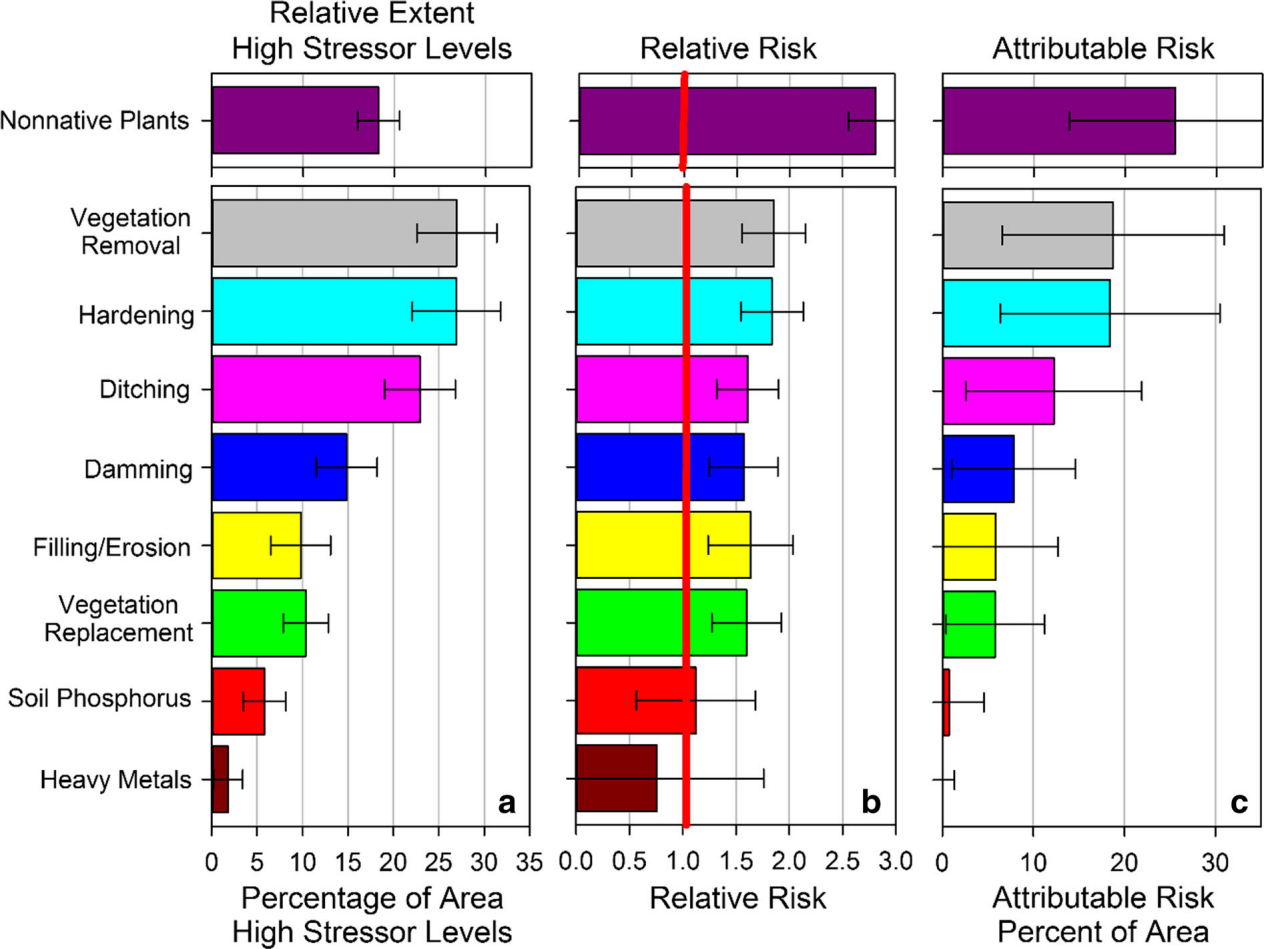
National estimates of **a** relative extent of stressor indicators occurring at high-stressor levels, **b** relative risk associated with each stressor indicator, and **c** attributable risk associated with each stressor indicator relative to wetland biological condition. Error bars are 95% confident intervals. The results from the nonnative plant indicator (NNPI) were added for information only because of the high interest in nonnative plants. The NNPI is not reported as part of the official NWCA risk results because plant data are used in the VMMI. See the “Estimating risk” section in the text for details (*adapted from* USEPA (2016a)

A national-scale analysis of risk from the NWCA-evaluated stressors provides an insight into potential management options. Three components of risk are considered for each indicator of stress: relative extent of wetland area with a high stressor-level (Fig. 8a), relative risk (Fig. 8b), and attributable risk (Fig. 8c).

The extent of stressors with high stressor-levels reported as part of the final report for the 2011 NWCA (USEPA 2016a) identified vegetation removal, hardening, and ditching as the stressors with the most wetland area in the conterminous US at high levels (Fig. 8a). Heavy metals and soil phosphorus had the least area at high-stressor levels.

The concept of relative risk addresses the question of severity of stressor effects on wetlands. It was adopted from the field of medicine because it is a powerful descriptor that is readily understood by most people (Van Sickle and Paulsen 2008). Paulsen et al. (2008) communicate this through an analogy with human health and the issue of heart disease. Specifically, they describe the case where one runs a greater risk of developing heart disease if one has high cholesterol levels. Often, such results are presented in terms of a relative risk ratio, e.g., the risk of developing heart disease is *X* times higher for a person with a high total cholesterol level than for a person with a low total cholesterol level. Relative risk for particular stressors to wetlands can be interpreted in the same way as the cholesterol example. For each of the evaluated indicators of stress, the relative risk value indicates how much more likely a wetland would be in poor biological condition if a stressor occurred at high stressor-levels than if the stressor occurred at low stressor-levels (Van Sickle and Paulsen 2008). A relative risk value of 1 or less indicates no association between the stressor and the biological indicator, whereas values greater than 1 suggest that high stressor-levels pose greater relative risk to biological condition. Confidence intervals for each relative risk ratio also were calculated. When the confidence intervals for any given ratio do not include 1, the relative risk estimate is statistically significant (Van Sickle and Paulsen 2008).

While the concepts of relative extent and relative risk are important taken alone, it is even more helpful to combine this information. Continuing the human example from above, high cholesterol levels might have a big impact on heart disease when they occur (i.e., relative risk), but if few people have high cholesterol levels (i.e., relative extent), little improvement in overall public health will be gained by focusing on reducing high cholesterol. The concept of attributable risk combines relative extent (how widespread) and relative risk (how big an impact) into a single number for each stressor (Van Sickle and Paulsen 2008). Attributable risk gives insight into how much of an improvement in wetland condition (as measured by the VMMI) might potentially occur if that specific stressor was reduced to zero (e.g., high stressor-levels reduced to moderate or low stressor-levels). Attributable risk can be compared across stressors to rank those that may be most problematic to wetland condition.

Figure 8 presents the results of the risk analysis nationally. Panel c is the focus when the interest is ranking stressors. For example, vegetation removal and hardening rank at the top of the list of stressors with roughly 18% attributable risk whereas soil phosphorus and heavy metals rank at the bottom (USEPA 2016a; Herlihy et al., 2019b). Interestingly, the relative risk (panel b) is roughly the same for all of the stressors (with the exceptions of soil phosphorus and heavy metals) (USEPA 2016a). The pattern in attributable risk then is almost entirely driven by the pattern in relative extent (panel a). In other words, when any of the top six stressors in panel c occur, they are likely to have a significant impact on wetland vegetation condition. But because the stressors vary dramatically in relative extent (i.e., how widespread high levels of each stressor occur), the potential management payoff of reducing the various stressors differs as seen in the attributable risk numbers.

Note that in Fig. 8, the results for the nonnative plant indicator of stress (NNPI) are separated from the other stressors. Where NNPI stressor-level is high or very high, the three risk measures that compose the indicator have large values, suggesting that nonnative plants strongly and negatively influence biological condition (USEPA 2016a; Magee et al. 2019b). The relative extent numbers clearly reflect how widespread high stressor levels related to nonnative vegetation are in wetlands. However, the relative risk numbers (and hence the attributable risk) may contain an analytical artifact that might overestimate impact related to the NNPI. The VMMI contains a metric that scores the relative importance (combination of frequency and cover) of native species. Two component metrics in the NNPI (relative nonnative frequency and cover) are potentially conversely related to the relative importance of native species metric in the VMMI. This means a site with high relative native species importance will also have low nonnative frequency or cover, with the converse being true. Consequently, the calculations for relative risk from NNPI will have some circularity for this indicator (USEPA 2016a; Magee et al. 2019b). Although nonnative species are a recognized threat to biological integrity of wetlands in the 2011 NWCA reporting, the use of relative and attributable risk analysis is not the best approach to evaluating the impact indicated by the NNPI.

## Uses of NWCA data to inform resource management and contribute to wetland science

The above description of the results from the 2011 NWCA demonstrates the comprehensive nature of the data that can be generated from monitoring and assessment, and suggests uses in resource management. As stated in Wardrop et al. (2013), reporting on the extent and condition of the resource can be used to track effectiveness of regulation and management practices by geographic region and/or wetland type. They further note that the estimates of the extent of stressors can identify the emergence of new threats to wetland condition, while the use of relative and attributable risk helps to prioritize management actions by stressor, geographic region, and/or wetland type.

In addition to the reporting of the NWCA results, there is an active research effort within NARS to develop additional ways to use the NWCA dataset and to explore interpretation of the data. For example, see Trebitz et al. (2019) on the use of water chemistry data as an indicator for the NWCA and Herlihy et al. (2019c) on the response of the NWCA indicators to various indicators of human disturbance at regional and continental scales.

Clearly, we believe that the NWCA provides valuable information to the USEPA, States, Tribes, Congress, and the public on our progress in maintaining and restoring wetland condition across the country. As the surveys continue, valuable information on trends will also emerge. We realize, however, that our intended use of NWCA data is not the only use. For example, the recent papers by Miller et al. (2016) on assessing condition of freshwater wetlands in the Northeastern US, Nahlik and Fennessy (2016) on carbon storage in US wetlands, Moon et al. (2017) on model extrapolation, and Stapanian et al. (2018) on land cover as a predictors of wetland vegetation quality used 2011 NWCA data. We invite our colleagues to access and use the NWCA data for exploring their own questions on wetland ecology and management. We welcome the insights that emerge from such independent analyses of the data.

## References

[CR1] Alloway BJ (2013). Heavy metals in soils: trace metals and metalloids in soils and their bioavailabilaity.

[CR2] Cowardin LM, Carter V, Golet FC, LaRoe ET (1979). Classification of wetlands and deepwater habitats of the United States.

[CR3] Dahl TE (2006). Status and trends of wetlands in the conterminous United States 1998 to 2004.

[CR4] Dahl TE (2011). Status and trends of wetlands in the conterminous United States 2004 to 2009.

[CR5] Dahl TE, Bergeson MT (2009). Technical procedures for conducting status and trends of the Nation’s wetlands.

[CR6] DeKeyser ES, Kirby DR, Ell MJ (2003). An index of plant community integrity: development of the methodology for assessing prairie wetland plant communities. Ecological Indicators.

[CR7] European Commission (2000). Directive 2000/60/EC of the European Parliament and of the Council of 23 October 2000 establishing a framework for community action in the field of water policy. *Official Journal of the European Communities, L327*, 1–72.

[CR8] Herlihy AT, Paulsen SG, Van Sickle J, Stoddard JL, Hawkins CP, Yuan LL (2008). Striving for consistency in a national assessment: the challenges of applying a reference-condition approach at a continental scale. Journal of the North American Benthological Society.

[CR9] Herlihy AT, Kamman NC, Sifneos JC, Charles D, Enache MD, Stevenson RJ (2013). Using multiple approaches to develop nutrient criteria for lakes in the conterminous USA. Freshwater Science.

[CR10] Herlihy, A. T., Kentula, M. E., Magee, T. K., Lomnicky, G. A., Nahlik, A. M., Serenbetz, G. (2019a). Striving for consistency in the National Wetland Condition Assessment: developing a reference condition approach for assessing wetlands at a continental scale. *Environmental Monitoring and Assessment*. 10.1007/s10661-019-7325-3. 10.1007/s10661-019-7325-3 PMC6586693 31222681

[CR11] Herlihy, A. T., Paulsen, S. G., Kentula, M. E., Magee, T. K., Nahlik, A.M., & Lomnicky, G.A. (2019b). Assessing the relative and attributable risk of stressors to wetland condition across the conterminous United States. *Environmental Monitoring and Assessment*. 10.1007/s10661-019-7313-7. 10.1007/s10661-019-7313-7 PMC6586707 31222378

[CR12] Herlihy, A. T., Sifneos, J. C., Lomnicky, G. A., Nahlik, A. M., Kentula, M. E., Magee, T. K., Weber, M. H., & Trebitz, A. S. (2019c). The response of wetland quality indicators to human disturbance across the United States. *Environmental Monitoring and Assessment*. 10.1007/s10661-019-7323-5. 10.1007/s10661-019-7323-5 PMC6586913 31222417

[CR13] Howarth W (2006). The progression towards ecological quality standards. Journal of Environmental Law.

[CR14] Kaufmann PR, Hughes RM, Van Sickle J, Whittier TR, Seeliger CW, Paulsen SG (2014). Lakeshore and littoral physical habitat structure: a field survey method and its precision. Lake and Reservoir Management.

[CR15] Lomnicky, G.A., Herlihy, A. T., Kaufmann, P. R. (2019). Quantifying the extent of human disturbance activities and anthropogenic stressors in wetlands across the conterminous United States – results from the National Wetland Condition Assessment. *Environmental Monitoring and Assessment*. 10.1007/s10661-019-7314-6. 10.1007/s10661-019-7314-6 PMC6586716 31222443

[CR16] Mack JJ (2007). Integrated wetland assessment program. Part 9: field manual for the vegetation index of biotic integrity for wetlands, v. 1.4.

[CR17] Mack JJ, Kentula ME (2010). Metric similarity in vegetation-based wetland assessment methods.

[CR18] Magee, T. K., Blocksom, K. A., & Fennessy, M. S. (2019a). A national-scale vegetation multimetric index (VMMI) as an indicator of wetland condition across the conterminous United States. *Environmental Monitoring and Assessment*. 10.1007/s10661-019-7324-4. 10.1007/s10661-019-7324-4 PMC6586711 31222469

[CR19] Magee, T.K., Blocksom, K. A., Herlihy, A. T., & A.M. Nahlik. (2019b). Characterizing nonnative plants in wetlands across the conterminous United States. *Environmental Monitoring and Assessment*. 10.1007/s10661-019-7317-3. 10.1007/s10661-019-7317-3 PMC6586712 31222487

[CR20] McCauley, D. J., Arnold, W. J., Saxton, J. B., & Turner, C. J. (2019). Applying adaptive management and lessons learned from national assessments to address logistical challenges in the National Wetlands Condition Assessment. *Environmental Monitoring and Assessment*. 10.1007/s10661-019-7320-8. 10.1007/s10661-019-7320-8 PMC6586718 31222449

[CR21] Miller KM, Mitchell BR, McGill BJ (2016). Constructing multimetric indices and testing ability of landscape metrics to assess condition of freshwater wetlands in the Northeastern US. Ecological Indicators.

[CR22] Moon JB, Dewitt TH, Errend MN, Bruins RJF, Kentula ME, Chamberlain SJ, Fennessy MS, Naithani KJ (2017). Model application niche analysis: assessing the transferability and generalizability of ecological models. Ecosphere.

[CR23] Nahlik, A. M., & Fennessy, M. S. (2016). Carbon storage in US wetlands. *Nature Communications*. 10.1038/nccomms13835. 10.1038/ncomms13835 PMC5159918 27958272

[CR24] Nahlik, A. M., Blocksom, K. A., Herlihy, A. T., Kentula, M. E., Magee T.K., and Paulsen, S.G. (2019). Use of national-scale data to examine human-mediated additions of heavy metals to wetland soils of the United States. *Environmental Monitoring and Assessment*. 10.1007/s10661-019-7315-5. 10.1007/s10661-019-7315-5 PMC6586720 31222398

[CR25] Olsen AR, Peck DV (2008). Survey design and extent estimates for the Wadeable Streams Assessment. Journal of the North American Bethological Society.

[CR26] Olsen, A. R., Kincaid, T. M., Kentula, M. E., & Weber, M. H. (2019). Survey design to assess condition of wetlands in the United States. *Environmental Monitoring and Assessment*. 10.1007/s10661-019-7322-6. 10.1007/s10661-019-7322-6 PMC6586691 31222669

[CR27] Omernik JM (1987). Ecoregions of the conterminous United States. Annals of the Association of American Geographers.

[CR28] Paulsen SG, Mayio A, Peck DV, Stoddard JL, Tarquinio E, Holdsworth SM, Sickle JV, Yuan LL, Hawkins CP, Herlihy AT, Kaufmann PR, Barbour MT, Larsen DP, Olsen AR (2008). Condition of stream ecosystems in the US: an overview of the first national assessment. Journal of the North American Bethological Society.

[CR29] Quétier F, Thébault A, Lavorel S (2007). Plant traits in a state and transition framework as markers of ecosystem response to land-use change. Ecological Monographs.

[CR30] R Core Team (2015). R: a language and environment for statistical computing.

[CR31] Scozzafava ME, Kentula ME, Riley E, Magee TK, Serenbetz G, Sumner R, Faulkner C, Price M (2011). The National Wetland Condition Assessment: national data on wetland quality to inform and improve wetlands protection. National Wetlands Newsletter.

[CR32] Shapiro MH, Holdsworth SM, Paulsen SG (2008). The need to assess the condition of aquatic resources in the US. Journal of the North American Bethological Society.

[CR33] Stapanian MA, Gara B, Schumacher W (2018). Surrounding land cover types as predictors of palustrine wetland vegetation quality in conterminous USA. Science of the Total Environment.

[CR34] Stevens DL, Jensen SF (2007). Sample design, implementation, and analysis for wetland assessment. Wetlands.

[CR35] Stevens DL, Olsen AR (1999). Spatially restricted surveys over time for aquatic resources. Journal of Agricultural, Biological, and Environmental Statistics.

[CR36] Stevens, D. L., & Olsen, A. R. (2000) Spatially restricted random sampling designs for design-based and model-based estimation. In *Accuracy 2000: Proceedings of the 4th International Symposium on Spatial Accuracy Assessment in Natural Resources and Environmental Sciences,* (pp. 609-616): Delft University Press, Delft.

[CR37] Stevens DL, Olsen AR (2004). Spatially-balanced sampling of natural resources. Journal of American Statistical Association.

[CR38] Stoddard JL, Larsen DP, Hawkins CP, Johnson PK, Norris RH (2006). Setting expectations for the ecological condition of streams: the concept of reference condition. Ecological Applications.

[CR39] The Conservation Foundation (1988). Protecting America’s wetlands: an action agenda. *Final Report of the National Wetlands Policy Forum*. Washington, DC: The Conservation Foundation.

[CR40] Trebitz, A. S., Nestlerode, J. A., & Herlihy, A. T. (NWCA Topical Collection). USA-scale patterns in wetland water quality as determined from the 2011 National Wetland Condition Assessment. *Environmental Monitoring and Assessment*. 10.1007/s10661-019-7321-7 PMC6638577 31222660

[CR41] USEPA (2011). Level III ecoregions of the continental United States (revision of Omernik, 1987).

[CR42] USEPA (2011). National Wetland Condition Assessment 2001: field operations manual.

[CR43] USEPA (2011). National Wetland Condition Assessment 2011: laboratory methods manual.

[CR44] USEPA (2011). National Wetland Condition Assessment 2011: site evaluation guidelines.

[CR45] USEPA (2016). National Wetland Condition Assessment 2011: a collaborative survey of the Nation’s wetlands.

[CR46] USEPA (2016). National Wetland Condition Assessment 2011: technical report.

[CR47] Van Sickle J (2010). Correlated metrics yield multimetric indices with inferior performance. Transactions of the American Fisheries Society.

[CR48] Van Sickle J, Paulsen SG (2008). Assessing the attributable risks, relative risks, and regional extents of aquatic stressors. Journal of the North American Benthological Society.

[CR49] Voulvoulis N, Arpon KD, Giakoumis T (2017). The EU Water Framework Directive: from great expectations to problems with implementation. Science of the Total Environment.

[CR50] Wardrop DH, Kentula ME, Stevens DL, Jensen SF, Brooks RP (2007). Assessment of wetland condition: an example from the Upper Juniata watershed in Pennsylvania, USA. Wetlands.

[CR51] Wardrop DH, Kentula ME, Brooks RP, Fennessy MS, Chamberlain S, Havens K, Hershner C, Brooks RP, Wardrop DH (2013). Monitoring and assessment of wetlands: concepts, case studies, and lessons learned. Mid-Atlantic freshwater wetlands: advances in wetlands science, management, policy, and practice.

[CR52] Whigham DF, Deller Jacobs A, Weller DE, Jordan TE, Kentula ME, Jensen SF, Stevens DL (2007). Combining HGM and EMAP procedures to assess wetlands at the watershed scale - status of flats and non-tidal riverine wetlands in the Nanticoke River watershed, Delaware and Maryland (USA). Wetlands.

